# Temporal patterns of gene expression in response to inoculation with a virulent *Anaplasma phagocytophilum* strain in sheep

**DOI:** 10.1038/s41598-023-47801-6

**Published:** 2023-11-21

**Authors:** Sveinung Eskeland, Erik G. Bø-Granquist, Snorre Stuen, Kari Lybeck, Peter Wilhelmsson, Per-Eric Lindgren, Shokouh Makvandi-Nejad

**Affiliations:** 1 Department of Production Animal Clinical Science, Faculty of Veterinary Medicine, Norwegian University of Life Sciences, Elizabeth Stephansens Vei 15, 1433 Ås, Norway; 2 Department of Production Animal Clinical Science, Faculty of Veterinary Medicine, Norwegian University of Life Sciences, Kyrkjevegen 332/334, 4325 Sandnes, Norway; 3 Norwegian Veterinary Institute, Elizabeth Stephansens Vei 1, 1433 Ås, Norway; 4 Division of Inflammation and Infection, Department of Biomedical and Clinical Sciences, Linköping University, 581 83 Linköping, Sweden; 5 Division of Clinical Microbiology, Laboratory Medicine, National Reference Laboratory for Borrelia and Other Tick-Borne Bacteria, Region Jönköping County, 553 05 Jönköping, Sweden

**Keywords:** Immunology, Bacterial infection

## Abstract

The aim of this study was to characterize the gene expression of host immune- and cellular responses to a Norwegian virulent strain of *Anaplasma phagocytophilum*, the cause of tick-borne fever in sheep. Ten sheep were intravenously inoculated with a live virulent strain of *A. phagocytophilum*. Clinical-, observational-, hematological data as well as bacterial load, flow cytometric cell count data from peripheral blood mononuclear cells and host’s gene expression post infection was analysed. The transcriptomic data were assessed for pre-set time points over the course of 22 days following the inoculation. Briefly, all inoculated sheep responded with clinical signs of infection 3 days post inoculation and onwards with maximum bacterial load observed on day 6, consistent with tick-borne fever. On days, 3–8, the innate immune responses and effector processes such as IFN1 signaling pathways and cytokine mediated signaling pathways were observed. Several pathways associated with the adaptive immune responses, namely T-cell activation, humoral immune responses, B-cell activation, and T- and B-cell differentiation dominated on the days of 8, 10 and 14. Flow-cytometric analysis of the PBMCs showed a reduction in CD4^+^CD25^+^ cells on day 10 and 14 post-inoculation and a skewed CD4:CD8 ratio indicating a reduced activation and proliferation of CD4-T-cells. The genes of important co-stimulatory molecules such as CD28 and CD40LG, important in T- and B-cell activation and proliferation, did not significantly change or experienced downregulation throughout the study. The absence of upregulation of several co-stimulatory molecules might be one possible explanation for the low activation and proliferation of CD4-T-cells during *A. phagocytophilum* infection, indicating a suboptimal CD4-T-cell response. The upregulation of *T-BET*, *EOMES* and *IFN-γ* on days 8–14 post inoculation, indicates a favoured CD4 Th1- and CD8-response. The dynamics and interaction between CD4^+^CD25^+^ and co-stimulatory molecules such as *CD28*, *CD80*, *CD40* and *CD40LG* during infection with *A. phagocytophilum* in sheep needs further investigation in the future.

## Introduction

The intracellular tick-borne rickettsial pathogen, *Anaplasma phagocytophilum*, causes tick-borne fever (TBF) in sheep and other domestic ruminants in Northern Europe as reviewed in^1^. In addition, the bacterium is considered to be an emerging zoonosis infecting humans, resulting in human granulocytic anaplasmosis^2^. The bacterium comprises several genetic variants which have been challenging to perform phylogenetic studies on^3, 4^. Tick-borne fever has severe impacts on animal welfare and the economy of the sheep industry^5, 6^. In sheep, TBF results in high fever (> 41 °C) followed by neutropenia 4–14 days post infection^7, 8^ that co-insides with initial bacteremia which can be monitored by quantitative polymerase chain reaction (qPCR) or by microscopy techniques^9, 10^. The neutropenia and bacteremia are most likely responsible for immune suppression resulting in secondary infections caused by bacteria such as *Staphylococcus aureus* or *Mannheimia haemolytica*. Infection with *Staphylococcus aureus* may result in arthritis, septicemia or abortions, while infection with *Mannheimia haemolytica* may result in pneumoniae^7, 11^. Pyrethroids are widely used in sheep to prevent tick infestation and transmission of tick-borne fever, and tetracyclines are used to treat infected individuals^12, 13^. Studies have shown that sheep may become infected despite the application of tick repellents, indicating that these treatments may be ineffective as prophylaxis^13, 14^. *Anaplasma phagocytophilum* has been shown to effectively evade host immune responses by using antigenic variation and incorporation in endosomal compartments of the host cells^7, 15, 16^. In addition, the bacterium is known to delay apoptosis of neutrophil granulocytes, as a strategy for bacterial propagation and persistence in the host organism^17–24^.

Several studies, primarily in cell culture systems, have generated knowledge of gene expression and gene regulatory mechanisms of the host cells in response to *A. phagocytophilum*^17, 20, 25, 26^. Previous studies have indicated that *A. phagocytophilum* infection results in proinflammatory responses^27–30^. There is a need for better prophylaxis against tick transmitted diseases in sheep and other animals. Several attempts have been made to develop effective vaccine candidates, by immunizing sheep with low virulent strains of the bacterium, formalin inactivated bacteria, Himar1-transformed bacterium or recombinant proteins^31–33^. Our objective was to study the immunological responses in sheep to *A. phagocytophilum*, ultimately resulting in an immunological profile of the host during the infection. An immunological profile is important in future vaccine work to understand the dynamics between the innate immune response and the adaptive immune response, such as the activation and proliferation of T- and B-cells.

## Material and methods

### Animals and premises

The research protocol and ethical considerations were approved by the Norwegian Animal Research Authority (protocol approval no. FOTS ID 8362 and FOTSID12093) upon formal application and in accordance with the EU Directive 2010/63/EU.

Fourteen sheep (*Ovis aries*) of the breed “Norwegian white sheep”, were selected from the research flock at the Norwegian University of Life Sciences (NMBU, Sandnes, Norway). Five rams and five ewes (ram 162–170 days old, ewes 199–211 days old) were inoculated with *A. phagocytophilum*, and four rams were used as negative controls (Supplementary Information S1). Due to rutting behaviour in young rams, these were separated from the ewes and intravenously inoculated 41 days before the ewes. The negative controls were included to control for any environmental- or feeding related issues during the trial and were monitored for clinical-, hematological-, and behavioral changes only. The sheep were confined indoors, in a tick free environment from birth and prior to the intravenous inoculation. Blood and serum from all individuals were evaluated by qPCR and immunofluorescence antibody test (IFAT) respectively, to exclude accidental previous exposure to *A. phagocytophilum*.

### Intravenously inoculation of *A. phagocytophilum*

**Figure 1:**
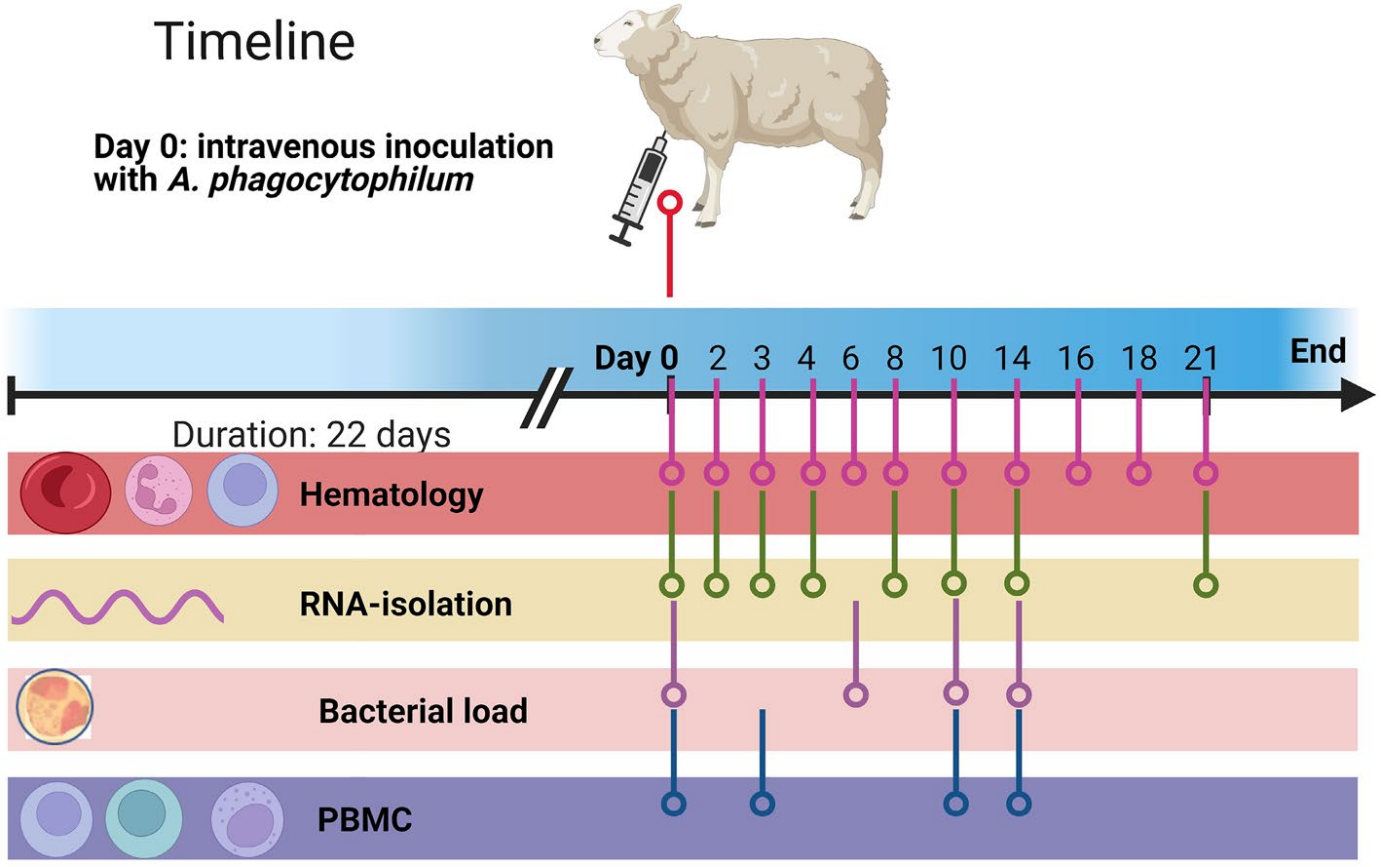
Timeline for the 22 day long study. All sheep were temperature measured daily (not shown in figure), hematology were assessed at eleven time points (tp) (0, 2, 3, 4, 6, 8, 10, 14 16, 18 and 21 dpi), samples for RNA-isolation collected on eight tp (0, 2, 3, 4, 8, 10, 14 and 21 dpi), samples for bacterial load on four tp (0, 6, 10 and 14 dpi) and sampling for flow cytometry of PBMC on four tp (0, 3, 10 and 14 dpi).

The inoculum was prepared by inoculation of infected sheep blood containing the Ap.Norvar1 16S-variant (GenBank acc.no M73220) to a naïve sheep (data not shown). After six days, a blood sample was assessed for bacterial load by microscopic evaluation of 400 neutrophil granulocytes in a May-Grünwald-Giemsa stained blood smear, and inoculation doses for ten sheep were prepared. The total inoculation dose of 0.4 ml donor blood contained approximately 1 × 10^6^ infected neutrophils and was transferred by intravenous injection through *vena jugularis* (*v. jugularis*) in the experimental animals. The control sheep were intravenously inoculated with an uninfected cell medium on day 0 (Supplementary Information S1, Fig. 1).

### Observational and sampling procedures

**Figure 2:**
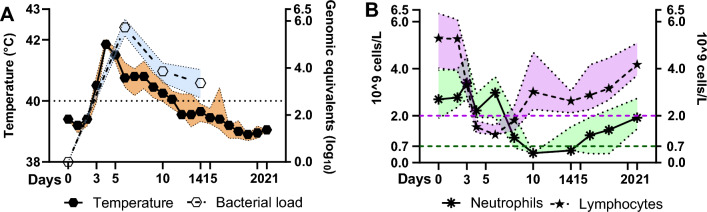
Temperature, bacterial load and hematological parameters. Data on bacterial load (Genomic equivalents, log_10_) (**A**, open pentagon, blue, right Y-axis), temperature (Celsius) (**A**, closed pentagon, orange, left Y-axis), lymphocyte count (**B**, five-pointed star, purple) (10^9^ cells/L), neutrophil granulocyte count (**B**, star, green) (10^9^ cells/L). The different symbols represent median observation for the respective time points, while the coloured areas represent the interquartile range (IQR) for the observations. Black stippled line in (**A**) describes upper limit for normal rectal temperature in sheep. Green stippled line and purple stippled in (**B**) describe the limits of neutropenia (0.7 × 10^9^ cells/L) and lymphocytopenia (2.0 × 10^9^ cells/L) in sheep. Data were analyzed with Repeated one way multiple comparison, further with Tukey’s multiple comparison test in GraphPad Prism (CA, USA). Significance p < 0.05, * < 0.05, ** < 0.01, * < 0.001. Control sheep are in Supplementary Fig. S1.

General clinical state and rectal temperature were observed daily during the study (Fig. 2). Blood samples were collected in vacutainers from *v. jugularis*. The following parameters were assessed (number of timepoints, tp, are in enclosed in brackets); hematology flowcytometry performed with ADVIA R 120 Hematology System (Erlangen, Germany) (eleven tp), bacterial load (four tp), peripheral blood mononuclear cells (PBMCs) cell count by flow cytometry (four tp) and transcriptomic-analysis (eight tp) (Fig. 2). Transcriptional analysis of blood and flow cytometry of PBMCs were not assessed in the four control sheep. The sheep that received *A. phagocytophilum*, functioned as their own controls in regard to the mRNA-analysis, with day 0 as the base line value. Two sheep were treated with one injection of flunixine meglumine (Flunixin ™) (Biovet, Quebec, Canada) on 7 dpi due to recumbency and reduced animal welfare. The medication quickly improved the clinical status of the sheep. We have chosen to include these two animals in the study since the injection was performed once, moreover a previous report of flunixine meglumine in sheep suggests a short half-life^34^. The hematological assays on 8 dpi differed not significantly from the other sheep, furthermore a previous in vitro study in bovine PBMCs did not observe reduced mRNA after addition of flunixine meglumine^35^, supporting our notion to include these animals in the study.

### Bacterial load quantification

Quantification of the total bacterial load was performed as previously described^32, 36^. In brief, extraction of DNA from 500 µL blood was performed on the MagNA Pure LC 2.0 instrument (Roche, Basel, Switzerland), using MagNA Pure LC DNA Isolation Kit—Large Volume (Roche), according to the manufacturer’s instructions. The bacterial load was quantified using a Taqman real-time PCR. Primers and a probe targeting the *A. phagocytophilum* citrate synthase gene (*gltA*). A pUC57 vector (Genscript USA Inc, NJ, US) carrying a region spanning the nucleotides 304–420 of the *gltA* gene (acc. no AF304137.1) were used for the standard curve preparation (Supplementary Table S1)^37^. The concentration of the plasmid solution was determined using a NanoDrop® ND-1000 instrument (Wilmington, DE, US). All samples were analysed in duplicates.

### Peripheral blood mononuclear cells isolation and flow cytometric analysis

Peripheral blood mononuclear cells were isolated according to Eskeland et al.^32^. The separation of PBMCs started within 8 h after sample collection. A density gradient medium (Lymphoprep; Axis-Shield, Norway) was used for separation of PBMCs from the other fractions of EDTA blood, as previously described^38^. Cells were subsequently stored at − 80 °C in a freezing solution consisting of FBS and 10% DMSO (Panreac Applichem ITV, Barcelona, Spain). Thawing and further analysis of PBMCs was in accordance with Eskeland et al.^31^. PBMCs were transferred to 96-well plates (3 × 10^5^ cells/well) and stained with LIVE/DEAD fixable Aqua Dead cell stain kit (Invitrogen, CA, USA), followed by incubation with unconjugated primary antibodies targeting selected surface markers and subsequently with the appropriate secondary antibodies (Supplementary Table S2). Compensation beads were used following manufacturer’s instructions (OneComp eBeads, eBioscience, San Diego, USA). The concentrations of monoclonal antibodies (mAbs) are shown in Table S2 in the Supplementary. A Novocyte flow cytometer (ACEA biosciences, San Diego, USA), and NovoExpress software, version 1.2.4 (ACEA biosciences, San Diego, USA) were used for cellular analyses. Absolute cell counts for the populations of CD4^+^, CD4^+^CD25^+^, CD8^+^, CD8^+^CD25^+^ were assessed during the study period. The gating was set to include mononuclear cells and exclude dead cells and doublets, gating strategy is presented in the Supplementary Fig. S3. The positive fluorescence gates were set with reference to negative control cells that were not stained with primary antibodies.

### RNA-isolation

For transcriptome analyses, whole blood was collected in Tempus RNA Fischer tubes (ThermoFischer Scientific, MA, USA) and stored at – 20 °C. Total RNA was extracted using Tempus Blood RNA Tube and Tempus Spin RNA Isolation kit in accordance with the manufacturer’s instructions (ThermoFischer Scientific, MA, USA). NanoDrop ND-1000 (Wilmington, DE, USA) was used for the quantification of the total RNA. Quality assessment of the RNA was done using Agilent BioAnalyzer and Agilent RNA 6000 Nano Kit in accordance with the producer’s protocol (Agilent Technologies, CA, USA). Samples with RNA integrity above the cut off-limit (RIN value > 7.0) were sent to the Norwegian Sequencing Centre (NCS, Oslo, Norway) for mRNA sequencing. Three samples did not meet the criteria and were not sent for mRNA sequencing (one sheep failed to reach the criteria on both day two and eight, another solely on day four).

### Transcriptome sequencing, assembly, annotation and statistical analysis

Transcriptome libraries were prepared by the Norwegian Sequencing Centre (NSC, Oslo, Norway) using a TruSeq® Stranded mRNA Library Prep kit, following the manufacturer’s protocol. Stranded sequencing was performed on Novaseq 6000 (Illumina, CA, USA) with paired end sequencing at 50 bp. Following read quality assessment by FastQC (www.bioinformatics.babraham.ac.uk/projects/fastqc/), Trimmomatic was used for quality assessment and trimming low quality bases in order to retain high quality reads^39^. Reads of low quality (Phred score < 30), of low complexity, with adapter sequences, or with sequences matching ribosomal or mitochondrial RNA, were discarded. Reads were mapped to the CRGh38/hg38 assembly using TopHat (version 2.0.13)^40, 41^ and reads with more than a single hit in the genome were discarded. Cufflinks^42, 43^ was used to generate transcriptome assemblies for each sequenced sample and all merged by Cuffmerge to construct a single gene transfer file. Expression data were normalized via the median of the geometric means of fragment counts across all samples, where relative expressions are expressed as fragments per kilobase of exon per million mapped reads (FPKM) values. Cuffdiff was then used to estimate the expression abundances of the assembled genes and transcripts and to test for differential levels of expression between groups. Genes or transcripts with > 1.5-fold difference in expression and corrected *p*-values (FDR adjusted) of < 0.05 were assigned as differentially expressed (DE). The figures illustrating the differentially expressed genes (DEGs) were plotted in RStudio (Version 1.4.1103).

### Functional enrichment and network analysis: gene ontology and pathway enrichment

Functional enrichment by assessing Gene ontology (GO) terms and pathway analysis, Kyoto Encyclopedia of Genes (KEGG) were carried out on our list of DEGs by the online tool String (https://string-db.org/) and plotted in RStudio. The analysis was performed against sheep reference genome with q-value < 0.005 (FDR, Benjamini and Hochberg method).

### Statistical evaluation

GraphPad Prism 9.1.0 (San Diego, CA, USA) was used in descriptive analyses of temperature, bacterial load, neutrophil granulocyte and lymphocyte count, as well as analyzes of cell count in CD4^+^, CD4^+^CD25^+^, CD8^+^ and CD8^+^CD25^+^. The Shapiro–Wilk test was used for evaluation of normality prior to mixed-effects analysis and Tukey’s multiple comparison test in data from CD4^+^, CD4^+^CD25^+^, CD8^+^, CD8^+^CD25^+^ and CD4:CD8 ratio. Significance level was set to 0.05.

## Results and discussion

The aim of the study was to investigate the immunological responses and gene expressions, viewed in concert with clinical, bacterial load and flow cytometry data in *A. phagocytophilum* inoculated sheep.

### *A. phagocytophilum* inoculated sheep developed increased bacterial load resulting in clinical signs of disease

The *A. phagocytophilum* inoculated sheep developed clinical signs of TBF in sheep as previously described^7, 9^. Rectal temperature equal to 40 °C or above, was defined as fever. The maximum body temperature was recorded on day 4 post inoculation (4 dpi) (maximum: 42.4 °C, median: 41.9 °C, interquartile range [IQR] 25%-75% [41.7–41.9 °C]) (Fig. 2A). Moreover, median duration of fever days was 8.5 (IQR 25–75%: [7.25–10.5]), median temperature was 40.8°C, which were in concordance with previous studies^7, 31, 44^. Maximum bacterial load developed on 6 dpi (maximum: 6.33 Log_10_, IQR 25%-75% [5.43–6.03], with a subsequent reduction on 10 and 14 dpi (Fig. 2A). The sheep developed lymphocytopenia on 4 dpi, this was also present on 8 dpi (Fig. 2B). Neutropenia was detected on 8–14 dpi (Fig. 2B). The negative control sheep did not develop clinical signs of infection, nor bacterial load or lymphocytopenia or neutropenia (as shown in Fig. S1 in Supplementary).

### General immunological pathways show an early innate immune response gradually shifting to an adaptive immune response eight days after inoculation

**Figure 3:**
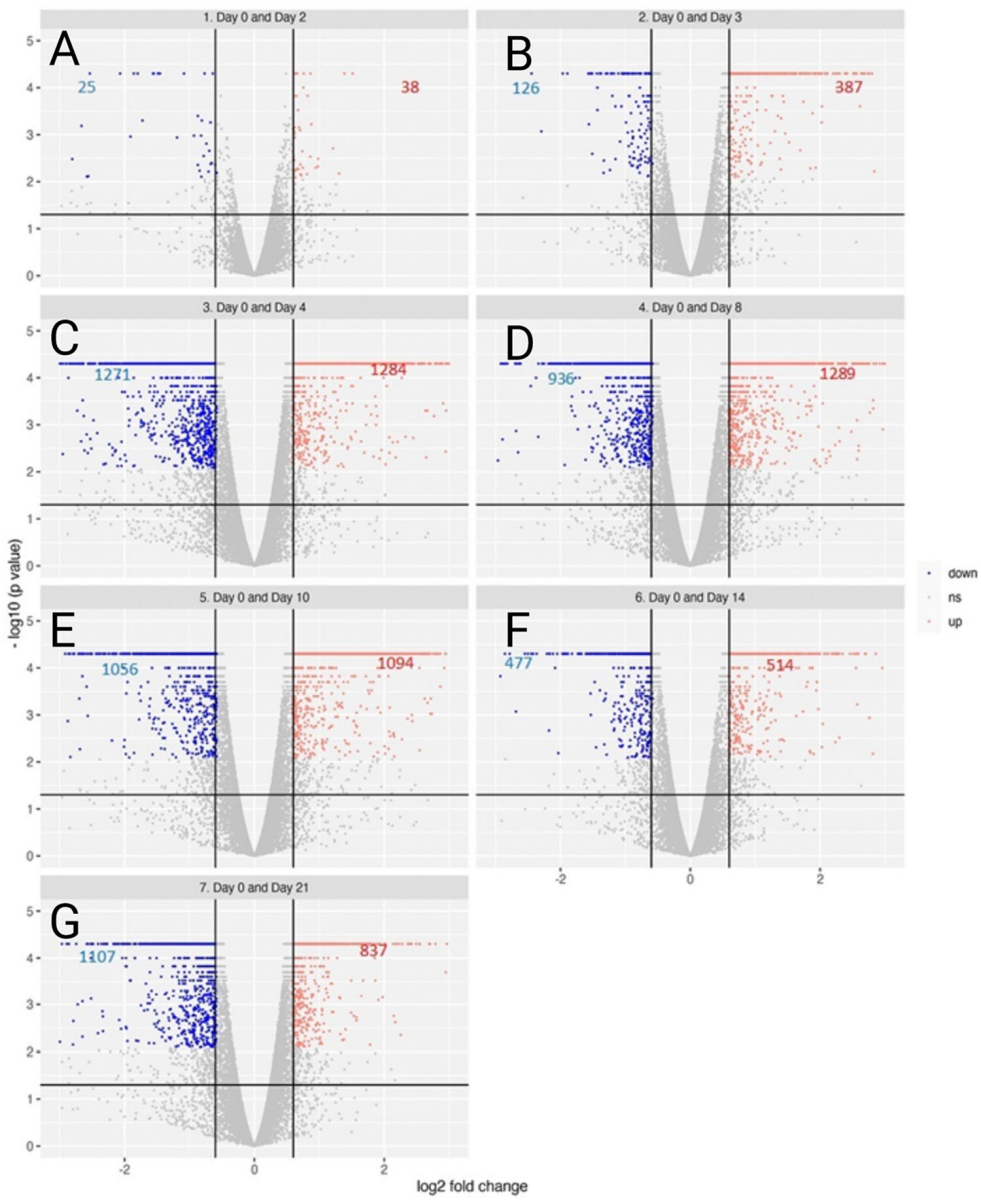
Gene expression profile shown in a volcano plot. All expression profiles were compared with expression profile obtained on day 0, prior to inoculation with *A. phagocytophilum*. The numbers of downregulated genes are represented in blue figures while upregulated genes are displayed in red, respectively. (**A**) Day 2. (**B**) Day 3. (**C**) Day 4. (**D**) Day 8. (**E**) Day 10. (**F**) Day 14. (**G**) Day 21.

A vast number of genes were significantly up- or downregulated throughout the study, here shown in volcano plots (Fig. 3). The levels of the gene expression were evaluated by comparing with the baseline, i.e., pre-inoculation (day 0). On 4 dpi, the maximum number of differentially expressed genes (i.e., significantly up- or downregulated; total number of genes: 2555 [upregulation: 1284, downregulation: 1271]) were identified (Fig. 3C).

**Figure 4:**
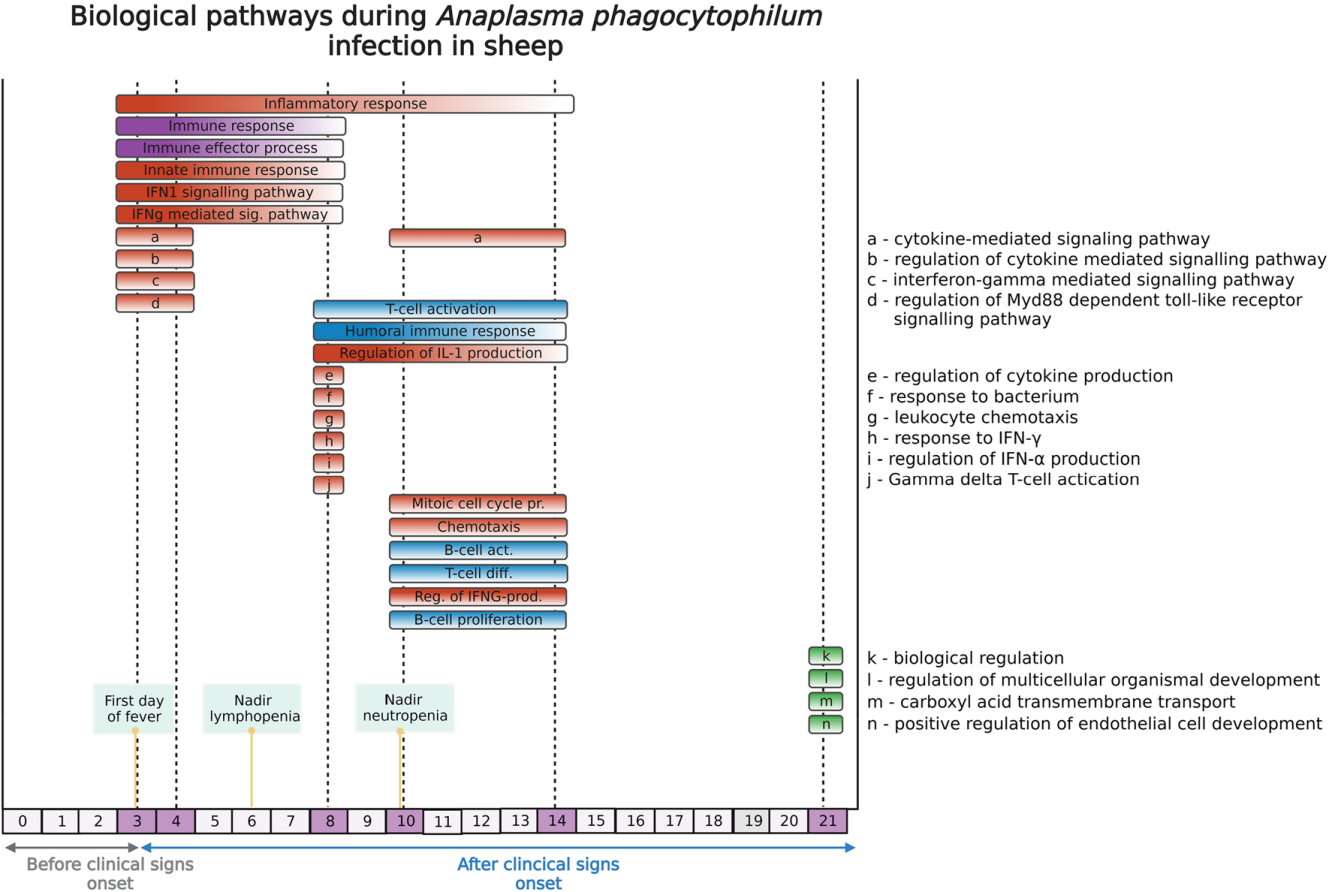
Biological pathways based on GO-numbers in context with neutropenia and lymphocytopenia observed in sheep. Red colour represents innate immune pathways, purple- general immune pathways, blue-adaptive immune system and green consist of various pathways. High intensity in colour reflects high gene ratio, low intensity (towards white) represents low gene ratio. The difference of intensity can only be compared within each pathway, and not against other pathways. Stippled vertical black lines as well as purple coloured areas on days show which days the sampling have been performed on (3, 4, 8, 10, 14 and 21). 14 pathways are listed with letters as follows; (**A**) cytokine-mediated signaling pathway, (**B**) interferon-gamma mediated signaling pathway, (**C**) regulation of Myd88 dependent toll-like receptor signaling pathway, (**E**) regulation of cytokine production, (**F**) response to bacterium, (**G**) leukocyte chemotaxis, (**H**) response to IFN-γ, (**I**) regulation of IFN-α production, (**J**) Gamma delta T-cell activation, (**K**) biological regulation, (**L**) regulation of multicellular organismal development, (**M**) carboxyl acid transmembrane transport, (**N**) positive regulation of endothelial cell development.

**Figure 5:**
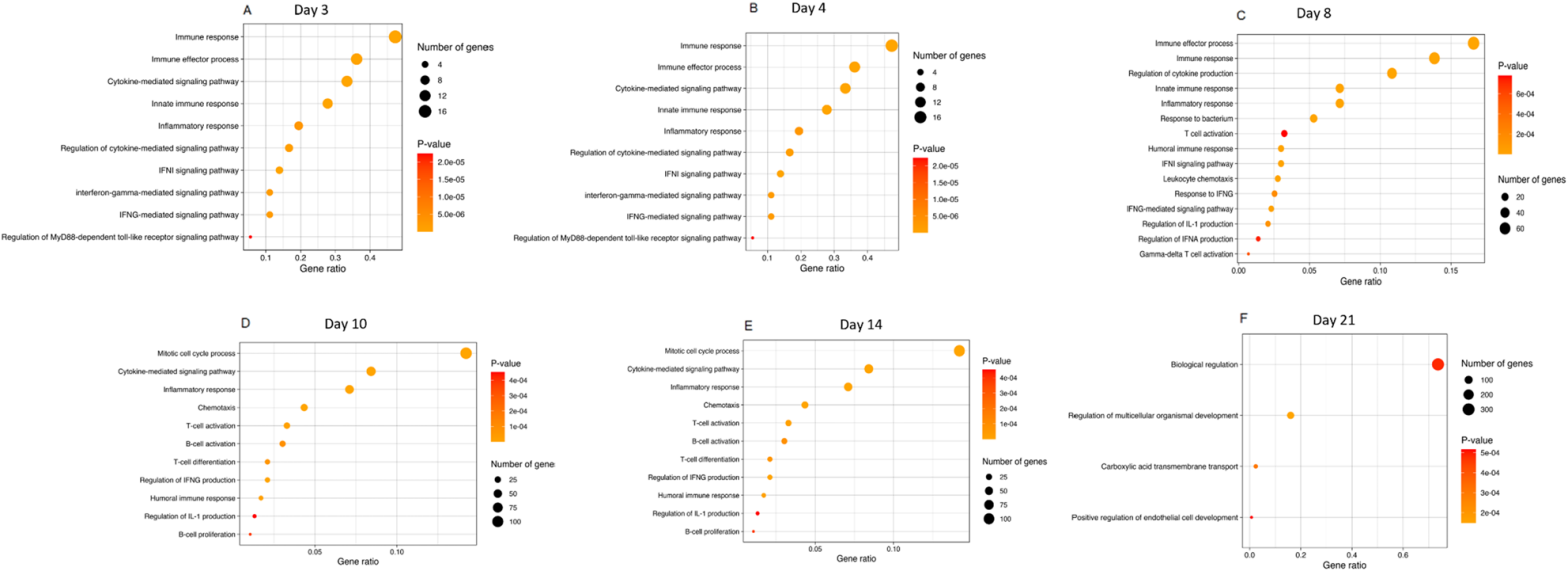
Biological pathways in GO-terms. Significance is described on a yellow–red colour scale, and the size of the circle reflects how many genes are associated with the respective pathway. (**A**) Day 3. (**B**) Day 4. (**C**) Day 8. (**D**) Day 10. (**E**) Day 14. (**F**) Day 21.

**Figure 6:**
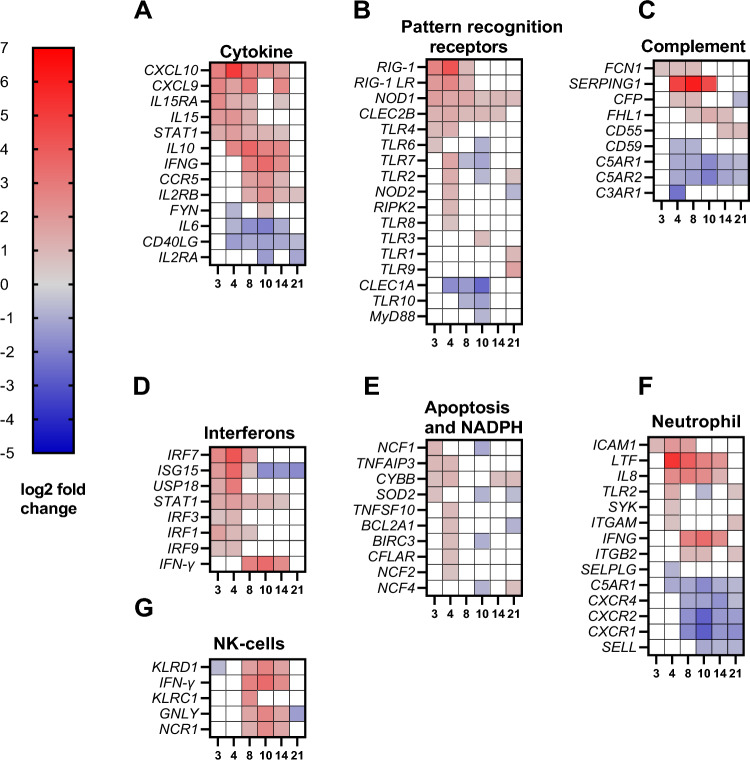
DEGs associated with innate immune responses. Results described in log2 fold change (blue-significantly downregulated, red-significantly upregulated). (**A**) Cytokine, (**B**) pattern recognition receptors, (**C**) complement, (**D**) interferons, (**E**) Apoptosis and NADPH, (**F**) Neutrophil, (**G**) NK-cells.

**Figure 7:**
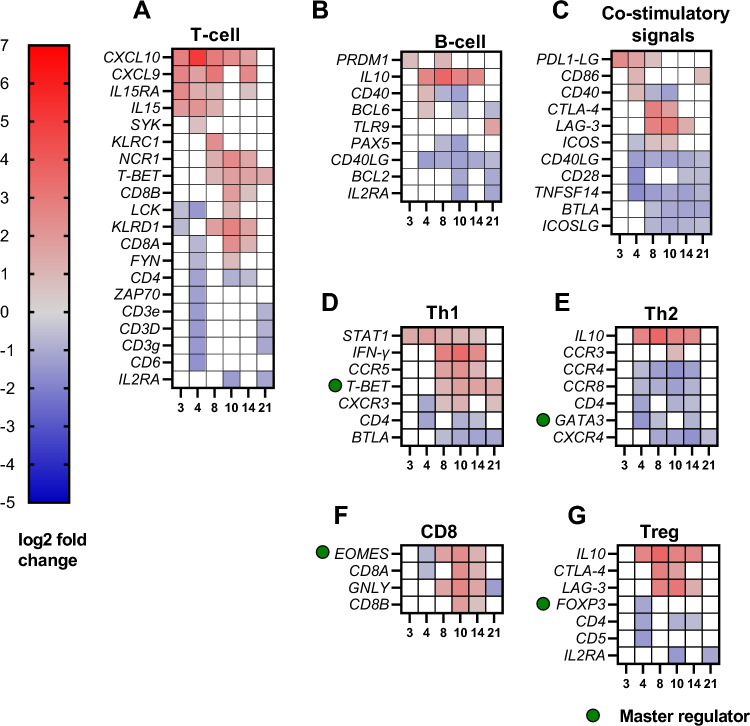
DEGS associated with adaptive immune responses. Results described in log2 fold change (blue-significantly downregulated, red-significantly upregulated). (**A**) T-cell, (**B**) B-cell, (**C**) Co-stimulatory signals, (**D**) Th1, (**E**) Th2, (**F**) CD8, (**G**) Treg. Genes assessed to be very important for regulation of a cell type, have been marked with “Master regulator” and a green circle.

Biological pathways, gene ontology annotations (GO-annotations) and DEGs were analysed. On 2, 3 and 4 dpi we observed responses associated with immune effector process, type IFN signalling pathway, IFN-γ-mediated signalling pathway and neutrophil activation (Figs. 4, 5, 6A–G and 7). Furthermore, on 3 and 8 dpi, DEGS associated with innate immune response were upregulated: cytokine-mediated signalling pathway and IFN-γ signalling (Figs. 4, 5 and 6A,D). On 8–14 dpi, the cellular and humoral immune responses were upregulated (Fig. 5), specific biologically pathways such as T-cell activation, humoral immune responses were prominent indicating a shift to an adaptive immune response (Figs. 4 and 5). DEGs associated with B- and T-cells, α and β T-cell activation and Th1- immune response were upregulated at the same time points (8–14 dpi) (Fig. 7D).

### Pattern recognition factors were dominated by upregulation of *RIG-1* and *NOD1*

Pattern recognition receptors (PRRs) are crucial in the innate immune response, including toll-like receptors (TLRs) which detect pathogens-associated molecular patterns (PAMPs) e.g. lipopolysaccharides (LPS) and peptidoglycans. However, *A. phagocytophilum* does not display LPS nor peptidoglycans due to absence of these genes^15^. Nonetheless, in the current study several genes associated with TLRs were upregulated on various time points, namely *TLR1* (21 dpi), *TLR2* (4 and 21 dpi), *TLR3* (10 dpi), *TLR4* (3 and 4 dpi), *TLR6* (3 dpi), *TLR7* (4 dpi), *TLR8* (4 dpi) and *TLR9* (21 dpi) (Fig. 6B). Downregulation of TLRs was observed for *TLR2* and *TLR6* on 10 dpi, and on 8 and 10 dpi for *TLR7* and *TLR10* and solely on 10 dpi for the TLR -associated gene *MyD88,* the latter being important in activation of the transcription factor NF-kB (Fig. 6B). Upregulation of *TLR*s during *A. phagocytophilum* infection was previously described by Galindo et al. (2008) who experimentally infected sheep with *A. phagocytophilum*, leading to increased *TLR7* expression in sheep buffy coat^27^. Borjesson et al. reported of upregulation of *TLR1* and *TLR2* genes, 24 h after in vitro infection of human neutrophils with *A. phagocytophilum*^17^. The impact of TLRs during *A. phagocytophilum* infection is not clearly established, and previous murine studies indicated that TLR2 and TLR4 signaling through MyD88, were not crucial for controlling the infection^45, 46^. However, previous in vitro studies^47, 48^ showed that infection with *A. phagocytophilum* resulted in activated macrophages and neutrophils capable of recognizing the bacterium via TLRs. In addition to upregulation of TLRs, we observed upregulation of *RIG1* (3–8 dpi), *RIG-1 LR* (3–8 dpi), *CLEC2B* (3–14 dpi) and downregulation of *CLEC1A* (4–10 dpi) which encode PRRs or PRRs associated structures (Fig. 6B). Furthermore, *NOD1* and *RIPK2* were upregulated during the infection on 3–21 dpi and 4 dpi, respectively. *NOD1* encodes an intracellular receptor and *RIPK2* encodes a receptor interacting protein important for NOD1 and NOD2, these were previously documented in infection studies with *A. phagocytophilum*^49, 50^ (Fig. 6B). Masumuto et al. (2008) showed that mice injected with a synthetic NOD1-ligand intraperitoneally resulted in increased neutrophil granulocyte recruitment into the murine peritoneal cavity^51^, suggesting that stimulated NOD1-receptors led to recruitment of neutrophil granulocytes. In our study, the neutrophil granulocyte count varied throughout the time period *NOD1* was upregulated (Fig. 2B) The brief upregulation of *NOD2* and *RIPK2* on 4 dpi might reduce the initial bacterial load, as previously documented in murine studies with *A. phagocytophilum* by Sukumuran et al. and Müller et al.^47, 50^, however the current study showed maximum bacterial load two days after on 6 dpi (Fig. 2A). To conclude, more studies will need to be conducted to understand the full role of PRRs role in the innate immune response against *A. phagocytophilum* infection.

### Downregulation of *SELPLG* observed two days prior to maximum bacterial load, questioning this ligand’s significance for *A. phagocytophilum* infection in sheep

Several ligands are important when neutrophils attach and adhere to endothelial cells during inflammation. In the current study we observed upregulation of genes important for cell-adhesion during inflammation, these were *ICAM1* (3–8 dpi), *ITGAM* (4 and 21 dpi) and *ITGB2* (the latter two comprise MAC- 1) (8, 10 and 21 dpi) (Fig. 6F). These results correlate with an in vitro study with human neutrophil granulocytes and endothelial cells infected with a human strain of *A. phagocytophilum*^52^. Furthermore, ITGAM and ITGB2 were reported to be important in the initial bacterial clearance of *A. phagocytophilum* in a murine study^53^. In the current study we were unable to determine if these genes were important in the initial clearance of the bacteria, moreover we noticed an upregulation of these genes in a late phase of the infection. During the study we observed other ligands important for adhesion, such as L-selectin and P-selectin glycoprotein ligand-1 (PSGL-1), encoded by *SELL* and *SELPLG* respectively. L-selectin is important for attachment of leukocytes to endothelial cells during inflammation as well as homing of T-cells to lymph nodes as reviewed in^54^. The observed downregulation of *SELL*, which was in a late phase of the infection (10–21 dpi) (Fig. 6F), might suggest a differentiation of memory T-cells and an activation of neutrophil granulocytes as reviewed in^54^. Moreover, a reduction in L-selectin in neutrophil granulocytes infected with *A. phagocytophilum* was previously reported by Choi et al., which supports the observed downregulation of this gene in the current study with regard to neutrophil granulocytes^52^.

PSGL-1 may adhere to endothelial cell by P-selectin or E-selectin, or interact with L-selectin on leukocytes as reviewed in^55^. Furthermore, the importance of *SELPLG*, encoding PSGL-1, was reported in previous studies where *A. phagocytophilum* attached to PSGL-1 and Sialyl-Lewis-X^56–58^, suggesting that the bacterium utilize this ligand to adhere and enter the host cell. We observed a downregulation of *SELPLG* on 4 dpi which is in line with Choi et al. (2003) who reported a reduction of PSGL-1 in neutrophils after heavy infection with *A. phagocytophilum* in an in vitro study^52^. A reasonable question is if downregulation of *SELPLG* results in reduced levels of bacteria in host cells? In the current study, we observed an increased bacterial load on 6 dpi as well as an increase in the neutrophil granulocyte population (Fig. 2B), even though *SELPLG* was downregulated on 4 dpi (Fig. 6F). Despite downregulated *SELPLG* on 4 dpi, we observed upregulation of *SYK* on the same day, the latter being important for PSGL-1 mediated infection by *A. phagocytophilum* (Fig. 7A)^59^. Whether the increase in bacterial load on 6 dpi was due to another entering or attachment mechanism than PSGL-1 or simply a result of increased bacterial propagation in host cells between 4 and 6 dpi we do not know. In light of the difference in regulation of *SELPLG* and *SYK* on 4 dpi we ought to consider several aspects, first: *A. phagocytophilum* does not utilize PSGL-1^60^ when the bacterium infects mice, second: several human strains of *A. phagocytophilum* have shown to have PSGL-1- independent strategies or receptors for infection of cells^61–63^. These aspects are worth considering and may suggest that ovine strains of *A. phagocytophilum* are utilizing other entering and adherence mechanisms than those previously described.

### *Anaplasma phagcoytophilum* infection led to down-regulation of receptors for C3 and C5 suggestive of a limited immune response

The complement system is important in the host’s innate and adaptive immune response through opsonization and killing of bacteria as well as triggering inflammatory responses as reviewed in^64^. However, the importance of complement during *A. phagcytophilum* infection remains uncertain, one study by Scorpio et al. showed that *A. phagocytophilum* increased its own propagation in a complement containing medium^65^. Presumably, the bacterium is only vulnerable for the complement system in the transitional stage between host cells. Complements, such as C3A and C5A, may also act as potent anaphylatoxins and chemoattractants in addition to being important in T-cell regulation during inflammation as reviewed in^64^. In the current study we observed no change in *C5*, while *C3* was excluded due to more than one observation for one time point (not shown). The reason for multiple observations of *C3* on one time point, is unknown. However, the genes responsible for the receptors of C3a and C5a, namely *C3AR1* (4 dpi), *C5AR1* (4–21 dpi) and *C5AR2* (4–21 dpi) (Fig. 6C), were downregulated throughout the infection. These receptors are crucial in complement-binding with C3a and C5a. Intriguingly, a previous in vitro study by Strainic et al. showed that a reduction in C3AR and C5AR in antigen presenting cells (APCs) reduced T-cell proliferation and differentiation^66^. Strainic et al. showed that upregulation of important genes for co-stimulation, such as *CD80*, *CD28*, *CD40* and *CD40LG*, were dependent on C3AR and C5AR receptors^66^. This may have implications for the immune response in sheep infected with *A. phagcocytophilum*, a possible consequence being a reduced immune response.

The upregulation of *FCN1*, encoding for the protein FICOLIN1 in the lectin pathway, was detected 3–8 dpi (Fig. 6C). Ficolin-1 is important in binding to PAMPS with sugar/glycan moieties on the bacterial surface as reviewed in^64^. The lack of LPS and peptidoglycans in *A. phagocytophilum*^15^ suggests that Ficolin-1 might bind to other glycan structures on the bacterium, a possible candidate might be the major surface protein 2 (MSP2), which undergo post transitional glycosylation^67^. In addition, Ficolin-1 is the only known human ficolin that can bind to sialic acids, e.g., Sialyl-Lewis-X, which suggests that this protein may play a role in the modulation of cell interaction in the immune system as reviewed in^68^. Two genes associated with negative regulation of the complement system were also regulated, namely *CD59* (downregulated 4–8 dpi) and *CD55* (upregulated 14–21 dpi) (Fig. 6C). These are negative regulators in the alternative and classical pathways of complement activation respectively, culminating in generation of the membrane attack complex (MAC) as reviewed in^64^. In regard to MAC, there is limited information on the importance of this in rickettsial infections, however Riley et al. argued that the complement’s proinflammatory responses were more important than MAC in in vivo studies with *Rickettsia australis*^69^. In our study, we observed downregulation of *CD59* suggesting less hindrance in formation of MAC, leaving target cells open for cell lysis and death as reviewed by^64^. The downregulation of *CD59* might be one explanation for the lymphocytopenia and the reduction in neutrophil granulocytes observed between 4 and 8 dpi (Fig. 2B). Furthermore, upregulation of *CD55* (14–21 dpi) ultimately limits cell lysis by limiting the classical and the alternative pathways (Fig. 6C). Complement factor properdin (*CFP*) (upregulated 4 and 8 dpi, downregulated 21 dpi) and Complement factor H (*FHL1*) (upregulated 8–14 dpi) encode a positive and a negative regulator of the alternative pathway, respectively as reviewed in^70^, suggesting that the alternative pathway was downregulated from 8 dpi (Fig. 6C). The downregulation of the complement system may be reasonable since the adaptive immune responses become more dominant during the later stages of the infection (Figs. 4 and 5C–E).

A crucial inhibitor of the complement system, C1-inhibitor, encoded by *SERPING1*, was strongly upregulated on 4–10 dpi (Fig. 6C). C1-inhibitor inhibits all the three pathways of complement system as reviewed by^71^. However, previous in vitro studies with *Borrelia recurrentis* and *Bordetella pertussis* showed these bacteria capable of binding to C1-inhibitor, resulting in serum resistance^72, 73^. An additional factor to be considered, is that the C1-inhibitor displays Sialyl-Lewis-X moieties on its surface, being one of the ligands that human strains of *A. phagocytophilum* uses when it adheres to neutrophil granulocytes^56, 74^. We cannot conclude on any interaction between C1-inhibitor and *A. phagocytophilum,* nor can we establish how the regulation of *C3AR1* and *C5AR2* impact the activation and regulation of T-cells. In order to achieve an understanding of the interaction between *A. phagocytophilum* and the complement system, more studies are needed.

### Several genes associated with anti-apoptosis were significantly upregulated four days after *A. phagocytophilum* inoculation

*A. phagocytophilum* renders neutrophil granulocytes a favorable environment for survival by thwarting the apoptotic process in human and sheep neutrophil granulocytes by the upregulation of anti-apoptotic genes that promote the extension of the cells' lifespan^17, 29, 49, 75^. In the current study, we observed the regulation of the antiapoptotic genes *TNFAIP3* (upregulated 3 and 4 dpi), *SOD2* (upregulated 3 dpi and downregulated on 10 and 21 dpi), *TNFSF10*, *BCL2A1* and *CFLAR* (all upregulated on 4 dpi) and *BIRC3* (upregulated on 4 dpi, downregulated on 10 dpi) (Fig. 6E). The pronounced upregulation of several of the anti-apoptotic genes on 4 dpi seems to have an immediate effect on neutrophil granulocyte count which increased on 6 dpi Furthermore, the highest bacterial load was detected on the same day (Figs. 2A,B, 6E). These results coincide with previous results for regulation of anti-apoptotic genes in *A. phagocytophilum* infected sheep^29^, supporting the notion that these genes are crucial for prolongation of the life span of neutrophil granulocytes in sheep as well as in humans.

### *CYBB*, a crucial component in the NADPH oxidative burst function of neutrophils, was upregulated on four out of six timepoints

In addition to the prolongation of the neutrophil granulocyte’s life span, *A. phagocytophilum* outmaneuvers the oxidative radicals of the cell^17, 76^. The genes *CYBB*, C*YBA, NCF1, NCF2 and NCF4* are important in the NADPH oxidative complex for the production of superoxide anion (O_2_^-^), this being important in neutrophil granulocyte oxidative burst during infections. Previous in vitro and in vivo studies; the latter in mice and sheep, showed a reduced oxidative burst post *A. phagocytophilum* infection^18, 77^. In the current study, *CYBB* (3, 4, 14 and 21 dpi) and *CYBA* (2 dpi not shown) were upregulated while the regulation of the other NADPH associated genes were accordingly: *NCF1* (upregulated 3 dpi, downregulated 10 dpi), *NCF2* (upregulated 4 dpi) and *NCF4* (downregulated 10 dpi, upregulated 21 dpi) (Fig. 6E). Previously, Woldehiwet et al. observed an increase in the gene expression of *CYBB* in *A. phagocytophilum* infected sheep^29^, while Borjesson et al. reported of an upregulation 24 h post incubation in human cells^17^. However, Banerjee et al. showed a reduction in *CYBB* in in vitro cell cultures infected with a human strain of the bacterium as well as in a murine model^77^. Our study supports the notion that there may be other factors than *CYBB* contributing to the reduction in oxidative burst within neutrophil granulocytes. Moreover, we observed a downregulation of *NCF1* and *NCF4* coinciding with neutropenia on 10 dpi (Figs. 2B. and 7E), this was in contrast to two previous studies with *A. phagocytophilum*, reporting of upregulation of the genes both in vitro and in vivo^17, 77^. We question if downregulated *NCF1* and *NCF4* may result in decreased oxidative burst during the late stages of the infection (Fig. 6E)? This is a valid question since sheep infected with *A. phagocytophilum* have shown to have reduced oxidative burst as long as three weeks post infection^18^.

### Regulation of neutrophil granulocyte associated genes suggest activation of these cells between 8 and 21 days after inoculation

In regard to neutrophil granulocytes, several chemokine receptors were downregulated on 8–21 dpi, namely *CXCR1*, *CXCR2* and *CXCR4* (Fig. 6F). Low levels of CXCR1 and CXCR2 expression are characteristic for activated neutrophil granulocytes as reviewed in^78^, suggesting that the neutrophil granulocytes are activated from 8 dpi and onwards (Fig. 6F). CXCR4 is a receptor for the CXCL12, a chemokine responsible for retention of neutrophil granulocytes in the bone marrow as reviewed in^79^. Decreased levels of *CXCR4* (8–21 dpi) may suggest that neutrophils are mobilized from the bone marrow and that the mobilization occurs from 8 dpi (Fig. 6F)^80^. This neutrophil granulocyte mobilization may not be sufficient to counter the neutropenia observed on 10 dpi since ruminants use 2–4 days to initiate a bone marrow response resulting in a peak response on day 4–7 as reviewed in^81^ (Fig. 2B). However, CXCR4 is also present on several other cell types besides neutrophil granulocytes, eg. T-cells, B-cells and monocytes as reviewed in^82^, which suggest some caution in the interpretation of this result. Indeed, neutrophil granulocytes are the dominating leukocyte in young ruminants, although our results show that the neutrophil cell count is lower than the lymphocyte cell count. This indicates a shift in the neutrophil granulocyte:lymphocyte ratio which is common for ruminants when they become mature (Fig. 2B) as reviewed in^81^.

*IL8,* an important chemoattractant for neutrophil granulocytes, was upregulated on 4–14 dpi (Fig. 6F), correlating with results in previous studies with *A. phagocytophilum* in mice, sheep and horses as well as in vitro with *A. phagocytophilum*^29, 30, 83–85^. Moreover, Klein et al. (1997) discussed if cytopenia seen during *A. phagocytophilum* infection could be caused by myelosuppressive actions caused by IL8, and if an increased pool of neutrophil granulocytes in peripheral tissue would increase bacterial propagation^85^. In the current study we observed an increase in bacterial load on 6 dpi, this occurred simultaneously with an elevated level of neutrophil granulocytes (Fig. 2B). This indicate that a larger pool of neutrophil granulocytes might result in increased bacterial load as previously hypothesized. IL8 activates neutrophil granulocytes through the receptors CXCR1 and CXCR2, subsequently leading to a reduction in CXCR1 and CXCR2 expression as reviewed in^78^. When neutrophil granulocytes become activated, they also become able to release the contents of their secondary granules^86^. Secondary granules consist of lactoferrin, encoded by *LTF* (upregulated 4–14 dpi) (Fig. 6F), and is anti-inflammatory and a modulator of the innate and the adaptive immune system^87^. Furthermore, LTF competes with IL8 in binding to glycoaminoglycans (GAGs) on endothelial cells during inflammation, which may diminish the recruitment of leukocytes to the inflammatory site^88^. Due to the association between IL8 and secondary granules, it is reasonable that *LTF* was regulated during the same time span as *IL8*. Thomas et al. implied a possible secondary granule deficiency due to *A. phagocytophilum* infection^89^, however the current study reports of upregulation of *LTF,* indicating production of secondary granules containing lactoferrin by neutrophil granulocytes during infection with *A. phagocytophilum*.

### Upregulation of IFN-γ was prominent between 8 and 14 days after inoculation with *A. phagocytophilum*

Interferon regulatory factors (IRFs) are important in the innate immune response due to their stimulation of interferon type 1 (IFN-α and IFN-β). These factors are mostly associated with viral infections, but in later years it has been acknowledged that they also occur in bacterial infections as reviewed in^90^. In the current study, upregulation of *IRF1* (3–8 dpi*)*, *IRF7* (3–8 dpi)*, IRF3* and *IRF9* (3 and 4 dpi) (Fig. 6D), suggest a strong stimulation of interferon regulatory factors, most likely resulting in a strong IFN-α and IFN-β response. A previous report by Colonne et al. showed upregulated IRF7 and IRF9 during an in vitro study with the intracellular bacterium *Rickettsia conorii*. The study implied that IRF7 and IRF9 were important because of their function in a positive feedback loop with IFN-β^91^. Furthermore, Thomas et al. observed decreased levels of IRF1 resulting in down-regulation of *CYBB* during an in vitro study with *A. phagocytophilum*, possibly resulting in a deficiency in NADPH oxidase activity^92^. However, our observations of upregulation of *IRF1* and *CYBB* on several timepoints are unable to support the hypothesis presented by Thomas et al.^92^ (Fig. 6D,E).

Several Interferon-stimulated genes (ISGs) were mainly upregulated on 3 and 4 dpi, ISG15 being one of the most prominent upregulated genes on 3–8 dpi, and highly downregulated on 10–21 dpi (Fig. 6D). The ISG15 is able to conjugate various proteins as a part of the innate immune response and has been most closely associated with antiviral immunity as reviewed in^93^. Moreover, ISG15 induce production of IFN-γ in lymphocytes and NK-cells, making the protein important during the immune response against virus such as HIV-1 in humans^94–96^. Previous studies indicate that several bacterial strains may be inhibited by the host’s upregulation of ISG15 as well; Colonne et al. reported of reduced levels of *Rickettsia conorii* in endothelial cell due to elevated levels of ISG15 and ubiquitin specific peptidase 18 (USP18)^97^, while Radoshevich showed that *Listeria monocytogenes* infection was restricted, when ISG15 was present^98^. However, we observed a peak in bacterial load on 6 dpi in our study despite upregulated *ISG15* between 3 and 8 dpi, questioning if ISG15 may result in decreased bacterial load of *A. phagocytophilum* in sheep (Figs. 2A and 6D)? Extracellular ISG15 may also be a potent inducer of IFN-γ in NK-cells and T-cells as reviewed in^93^, although in our study concurrent upregulation of *ISG15* and *IFN-γ* was only detected on 8 dpi (Fig. 6D). The impact of *ISG15* on *A. phagocytophilum* infection should be addressed, since we know that it clearly impacts a wide variety of other intracellular bacterial infections^96–98^.

The cytokine IL6 is highly pleiomorph and is deemed important in the development of several autoimmune and chronic inflammatory diseases in humans such as rheumatoid arthritis and systemic lupus erythematosus as reviewed in^99^. In the current study we observed downregulation of *IL6* on 4–14 dpi (Fig. 6A), which is in line with a previous *A. phagocytophilum* infection study in horses where peripheral blood monocytes were assessed^83^. However, in vitro studies on human peripheral blood monocytes infected with *A. phagocytophilum* showed elevated mRNA levels of *IL6*^100, 101^, furthermore elevated levels of *IL6* mRNA were reported from sheep mononuclear cells in an in vivo study with *A. phagocytophilum*^29^. The discord between these results are most likely due to the difference in the sample material and procedure such as transcriptomics of whole blood compared to peripheral blood monocytes. We suggest that IL6 is not a strong contributor to a possible proinflammatory response in sheep, but that this predominantly is a result of IFN-γ, which is reported in several species infected with the bacterium^29, 30, 102^.

We observed a significant upregulation of *IFN-γ* on 8–14 dpi (Fig. 6D). In the same time period, the bacterial load consistently decreased on 10 and 14 dpi (Fig. 2A), supporting that IFN-γ has a negative effect on the propagation of the bacteria as previously suggested, although IFN-γ was not deemed critical for clearance of the bacterium^103–105^. Previous reports from humans, sheep, horses and mice studies showed elevation in the levels of IFN-γ, resulting in discussions whether the elevated levels of IFN-γ was the cause of immunopathology observed in human cases^29, 83, 84, 105–107^. Several studies have suggested that it is not IFN-γ alone resulting in immunopathology in humans, but rather a skewed balance beneficial for IFN-γ compared with IL10. The dynamic between IFN-γ and IL10, an anti-inflammatory cytokine, has previously been described in a murine study by Martin et al.^107^. In the current study, *IL10* was upregulated 4–14 dpi (Fig. 6A) suggesting that this would balance the effects of proinflammatory cytokines such as IFN-γ as reviewed in^108^. Furthermore, IL10 is also considered to inhibit upregulation of important co-stimulation molecules on dendritic cells as well as resulting in decreased IL2 production resulting in impaired T-cell responses, supporting the notion of an impaired adaptive immune response due to infection with *A. phagocytophilum*^109, 110^.

Innate immune cells, such as NK-cells, produce IFN-γ as a part of the host immune response^111^. It is therefore of particular interest to study how genes important for activation of NK-cells are regulated during infection with *A. phagocytophilum*. In the current study *KLRD1* (downregulated 3 dpi, upregulated 8–14 dpi), encodes a receptor that may interact with the NK-inhibitory *KLRC1* (upregulated 8 dpi), *KLRC2 or KLRC3* (neither were significant regulated-not shown), suggesting a brief inhibition of NK-cells on 8 dpi (Fig. 6G). However, the *NCR1* (NKp46), which is a marker for the rested and activated ovine NK-cells^112^, was upregulated 8–14 dpi suggesting an increased response by NK-cells coinciding with previous mentioned upregulation of *IFN-γ* on 8–14 dpi (Fig. 6G). Furthermore, the suggested increase in response by NK-cells on 8–14 dpi, is supported by the upregulation of *GNLY* on 8–14 dpi (Fig. 6G). The gene *GNLY* encodes the cytotoxic granulysin secreted by cytotoxic T-cells and NK-cells, which has proved to be important against intracellular infections as reviewed in^113^.

### Decreased levels of CD4^+^CD25^+^-T-cells suggest reduced activation of CD4 T-cells

The chemokines and interleukins *CXCL9*, *CXCL10* and *IL15* were upregulated from 3 dpi ending on 14 or 8 dpi (Fig. 6A). CXCL9 and CXCL10 are chemokines that target the CXCR3 receptor (downregulated 4 dpi, upregulated 8, 10 and 21 dpi) (Fig. 7D), the latter being highly expressed on activated and effector T-cells such as CD4 Th1 and CD8-cells as reviewed in^114^, although they also appear in lymphoid cells, dendritic cells, NK-cells and some subsets of B-cells as reviewed in^78^. The upregulation of CXCR3 support activation of T-cells on these time points.

**Figure 8:**
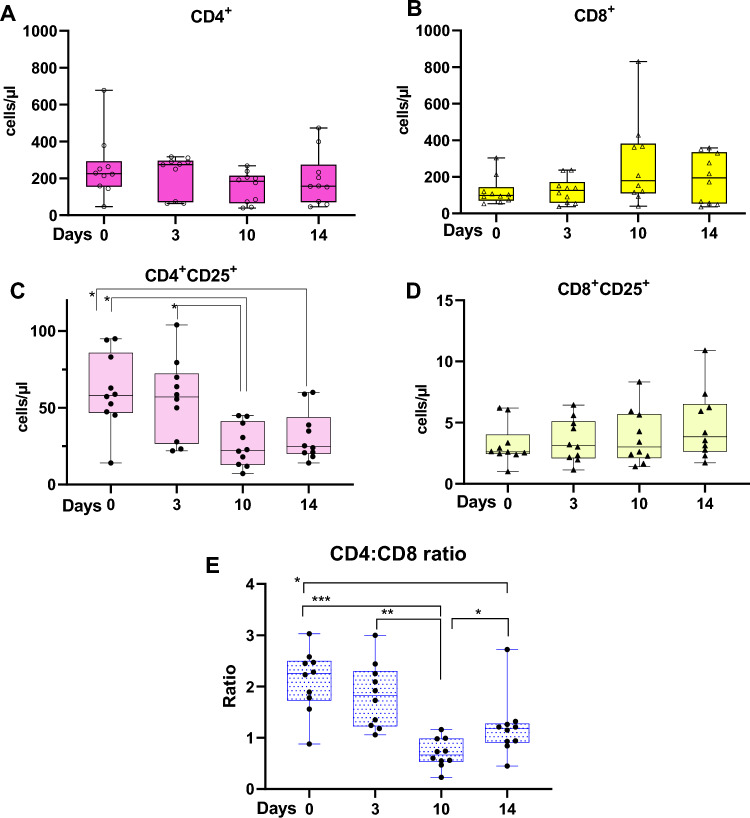
PBMC flowcytometry. Total cell count on four time points (days 0, 3, 10 and 14). (**A**) CD4^+^ T-cell count (open circle, pink), (**B**) CD8^+^ T-cell count (open triangle, yellow), (**C**) CD4^+^CD25^+^ T-cell count (closed circle, light pink), (**D**) CD8^+^CD25^+^ T-cell count (closed triangle, light yellow. (**E**) CD4 ^+^CD8^+^ ratio. Data were analyzed with Repeated one way multiple comparison, further with Tukey’s multiple comparison test in GraphPad Prism (CA, USA). Significance p < 0.05, * < 0.05, ** < 0.01, * < 0.001. Respective coloured area represent the IQR for the observations.

Several cytokines were regulated throughout the study, one being the IL15 which is critical for the survival of CD8-memory cell and promotion of NK-cell proliferation as as reviwed in^115^. IL15 is also important for induction of the previously mentioned granulysin (*GNLY*) from cytotoxic T-cells and NK-cells (Fig. 7F), although in the current study there was one day of overlap in upregulation of the two genes, *IL15* (3–8 dpi) and *GNLY* (8–14 dpi) (Figs. 6A and 7F). The gene to the respective receptor of IL15, *IL15RA*, was upregulated 3–8 dpi as well as 14 dpi (Fig. 6A). Genes associated with IL15 receptor were *IL2RB* (upregulated 8–21 dpi) and *IL2RG* (no significant regulation, not shown), indicating the importance of IL15 during the infection (Fig. 6A). The subunits *IL2RB* and *IL2RG* are shared with the receptor of cytokine IL2, which share some of the functions to IL15, although IL2 are predominantly more important in maintenance of the T-regulatory-cell (Treg-cell) population and in activation-induced cell death (AIDC) as reviewed in^115^. Furthermore, IL2 is also crucial in activation and proliferation of T-cells into effector and memory cells as reviewed in^115, 116^, moreover previous studies with *A. phagocytophilum* infected sheep reported of reduced proliferation of lymphocytes after stimulation with mitogens^117, 118^. There are contradictory results regarding the expression of *IL2* in studies with *A. phagocytophilum*; two studies with sheep and horses^27, 83^ reported of no significant change of *IL2,* while Woldehiwet et. al (2014) reported of significant upregulation of the gene in *A. phagocytophilum* infected sheep^29^. In our study, we neither observed an up- or downregulation of *IL2*. Moreover, the receptor of IL2 consist of the previously mentioned IL2RB and IL2RG, in addition to the unique receptor IL2RA (CD25) which is a marker for activated T-cells as well as Treg-cells as reviewed in^115^. In a previous study by Galindo et al. *IL2RA* was upregulated in buffy coat from two experimental infected sheep on day 6–9 dpi and downregulated in naturally infected sheep, however no flow cytometry data on PBMCs, more specific CD4^+^CD25^+^ cells were presented^27^. In our study we observed a downregulation of *IL2RA* (*CD25*) (10 and 21 dpi) (Fig. 6A). Indeed, our results from flow cytometric analysis of PBMCs, show a reduction in the CD4^+^CD25^+^ cell population; 0–10 dpi (p = 0.0003), 0–14 dpi (p = 0.0223), and 3–10 dpi (p = 0.0036) (Fig. 8C). The reduction in CD4^+^CD25^+^ cell population correlates with a downregulation of *CD25* (*IL2RA*) indicating a reduction in CD4-T cell activation and proliferation on 10 dpi. The reduction in CD4^+^CD25^+^ cell population coincides with Whist et al. who reported of a significant reduction in CD4^+^CD25^+^ T-cells percentage in sheep infected with *A. phagocytophilum*^119^. Furthermore, our study observed downregulation of *CD4* on 4, 10 and 14 dpi (Fig. 7A). The lack of upregulation of *IL2*, down-regulation of *IL2RA* and the reduction in CD4^+^CD25^+^ cell population suggest a reduced activation and proliferation of CD4-cells in the early stages of the adaptive immune response. The impact of a reduced CD4-cell response during *A. phagocytophilum* infection in sheep will most likely restrict the host’s abilities to eliminate the bacteria. This may be one reason for why the bacterium is able to persist in the host, since a previous study have reported that CD4-cell response is crucial for eliminating the bacterium^104^.

### Key genes in T- and B-cell activation and proliferation were downregulated suggesting a sub-optimal adaptive immune response

Activation of T-cells occurs through the TCR-signaling pathway involving LCK and FYN as central members in the Src kinases family as reviewed in^120^, but also ZAP-70 is important in this context. In the current study, *ZAP-70* (downregulated on 4 dpi), *LCK* (downregulated 3 and 4 dpi, upregulated 10 dpi) and *FYN* (downregulated 4 dpi, upregulated 10 dpi) indicate activation of T-cells on 10 dpi (Fig. 7A). The absence of upregulation of these genes on 8 or 14 dpi might be normal, but it is worth mentioning that reduced LCK has resulted in reduced production of IL2 from T-cells as reviewed in^120^. Furthermore, the absence of IL2 has been reported in several previous studies with *A. phagocytophilum* as well as in the current one^27, 83^, which makes it relevant to study how *LCK* and *FYN* are regulated during *A. phagocytophilum* infection in sheep.

Activation of T-cells are further supported by upregulation of the co-stimulation genes *ICOS* on 8 dpi and 10 dpi and *CD86* on 4 and 21 dpi (Fig. 7C). Moreover, the ICOS ligand, (*ICOSLG*), observed on APCs such as resting B-cells, dendritic cells and activated monocytes, was downregulated between 8 and 21 dpi (Fig. 7C) and *CTLA-4* was upregulated (8 and 10 dpi) suggesting an activation of T-cells (Fig. 7C) as reviewed in^121, 122^. This notion is further supported by the downregulation of *BTLA* on 8–21 dpi, an important co-stimulation molecule for T- and B-cells as reviewed in^123^ (Fig. 7C). Further, we would like to emphasize that both CTLA-4 and BTLA act as immune checkpoints for T-cells by transmitting inhibitory signals limiting effector T-cell function as reviewed in^123, 124^, advocating some caution when interpreting the impact of these genes in the current study.

In contrast to the abovementioned results on *CTLA-4* and *BTLA*, the down-regulation of *TNFSF14* (4–21 dpi) suggest an insufficient activation and proliferation of T-cells since this receptor is upregulated on activated T-cells as reviewed in^125^ (Fig. 7C). This is supported by the downregulation of *CD6* (4 dpi) which is important in activation and proliferation of T-cells^126, 127^, although upregulation was solely observed on 4 dpi (Fig. 7A). Furthermore, the most important co-stimulation molecules such as *CD28* (downregulated 4 dpi and 14–21 dpi), *CD80* (no significant alteration, not shown), and *CD3e, CD3d and CD3g* (downregulated dpi 4 and 21), were predominantly downregulated (Fig. 7A), suggesting a suboptimal T-cell response. One of the most interesting results was the significant downregulation of *CD40LG* on 4–21 dpi, the ligand being important in cellular and humoral immunity (Fig. 7C). Indeed, CD40LG is important in activation of cytotoxic response in CD8-cells, but it is also imperative in humoral immunity by stimulating the process of antibody isotype switching and affinity maturation, ultimately resulting in memory B-cells and long-lived plasma cells^128–130^. A previous study by Birkner et al. (2008) reported that mice deficient for CD40, the receptor of CD40LG, developed increased bacterial load of a human strain of *A. phagocytophilum* and were unable to clear the infection^104^. Moreover, several studies have reported of decreased antibody titres in sheep infected with *A. phagocytophilum* during simultaneously vaccination against other agents^118, 119, 131^. An intriguing question is if the downregulation of *CD40LG* might be one of the reasons for the abovementioned reduction in humoral response, as well as being instrumental in the subsequent immune suppression leaving sheep susceptible to secondary pyogenic infections?

Studies performed on chronic infections, showed upregulation of LAG3 and PDL1, these being associated with T-cell exhaustion resulting in the loss of effector functions e.g. IL2 production and a reduction in proliferative and cytotoxic capabilities as reviewed in^132^. In a previous study by Okagawa et al., the author reported of upregulation of PDL1 and LAG3 on PBMCs in *Anaplasma marginale* infected heifers during the acute phase of the infection^133^. Okagawa showed that antibodies blocking PDL1 and LAG3 receptors resulted in increased T-cell function, thus suggesting that PDL1 and LAG3 may impact T-cell function in *A. marginale* infected heifers^133^. In the current study, we observed upregulation of *LAG3* on 8–14 dpi and the ligand of PDL1; *PDL1-lig* (4–8 dpi), although no difference was observed for *PDL1* (Fig. 7C). The upregulation of these genes viewed in context with the absence of upregulation of *IL2*, is suggestive of a possible restricted T-cell response. To conclude, the current study warrants increased focused on co-stimulatory molecules present on PBMCs from *A. phagocytophilum* infected sheep in order to understand the dynamics of T-cell response.

### Genes associated with Th1- and CD8-responses become predominant in the adaptive immune response

In our results, with regard to genes associated with B-cells, the downregulation of *BCL6* on 10 and 21 dpi and *PAX5* on 8 and 10 dpi (Fig. 7B), suggest activation and differentiation of B-cells on 8 and 10 dpi^134^. This inference can be explained since previous studies showed that BCL6 controls PRDM1 regulation (upregulated 3 and 8 dpi) (Fig. 7B), the latter being an important transcription factor resulting in plasma cell differentiation of B-cells by restricting *PAX5* regulation^134, 135^. However, the previous mentioned downregulation of co-stimulatory genes such as *CD40* and *CD40LG* would most likely have a negative impact on activation and proliferation of B-cells (Fig. 7C). Future studies should investigate these gene’s interplay in order to determine which of them make the greatest impact on activation and differentiation of B-cells during an *A. phagocytophilum* infection in sheep.

Beside NK-cells, CD4 T-helper 1 cells (Th1-cells) are important for production of IFN-γ and a proinflammatory response. One important transcription factor in the Th1-immune response is *STAT1* (upregulated 3–14 dpi), which is deemed important in regulation of IFN-α, IFN-β and IFN-γ^136^ (Fig. 7D). In a previous infection study with *A. phagocytophilum* by Choi et al., the author reported of a significant correlation between STAT1 signalling and IFN-γ^137^. This correlation was supported by our observation of simultaneously upregulation of *STAT1* and *IFN-γ* (8–14 dpi) (Fig. 7D). Furthermore, Choi et al. showed that mice deficient for IFN-γ, but with intact STAT1 signalling, developed increased bacterial load and unexpectedly reduced proinflammatory immune response^138^. The author suggested that STAT1, besides inducing IFN-γ production, also were capable of reducing inflammation by an unknown mechanisms^138^. STAT1 is also important by inducing the transcription factor, *T-BET*, which induce differentiation of Th1-cells^139^. *T-BET* was upregulated on 8–21 dpi (Fig. 7D) indicating a proinflammatory immunological response corresponding with the action of CD4 Th1-cells^140^. Moreover, T-BET is capable to downregulate *GATA-3*, an important transcription factor in the Th2 immune response^139^. In our study *GATA-3* was downregulated 4–8 dpi and 14 dpi, suggesting a down-regulation of the Th2-immune response on these time points. (Fig. 7E)^139^. This notion is further supported by the downregulation of genes encoding CCR4 and CCR8 receptors (downregulated on 10 dpi and 4–14 dpi respectively) which are deemed important in Th2-immune response^141^ (Fig. 7E). On the other hand, *CCR3*, another receptor in the Th2-immune response was upregulated, although this was only briefly on 10 dpi (Fig. 7E), while the Th1-receptor associated gene *CCR5* was upregulated on 8–14 dpi^142^ (Fig. 7D). To conclude, the observed upregulation and downregulation of genes associated with a Th1- or a Th2 immune response respectively, support the idea that sheep respond with a Th1-immune response against *A. phagocytophilum* infection. This is in accordance with previous studies in *Anaplasma* spp. and *Rickettsia* spp. which have reported of a predominant Th1-immune response^30, 143^.

The transcription factor *FOXP3,* which is deemed important for development and function of regulatory T-cells, was downregulated on 4 dpi (Fig. 7G) as reviewed in^144^. Interestingly, this gene was not further upregulated during the infection (Fig. 7G). The absence of upregulation on consecutive time points leads us to question the strength of T-cell regulatory response and whether this impacts the balance between proinflammatory and anti-inflammatory responses in the host as reviewed in^144^. This notion may be supported by our previously reported low numbers of CD4^+^CD25^+^ cells in the section “Decreased levels of CD4^+^CD25^+^- T-cells which suggests a reduced activation of CD4 T-cells”, although these results include both the effector and T-regulatory cells (Fig. 8C). There is limited information on the impact of CD4^+^CD25^+^ FoxP3 cells on *A. phagocytophilum* infections, however an infection study with *Rickettsia conorii* by Fang et al. reported of decreased levels of CD4^+^ Foxp3^+^ cells from murine spleen^143^. However, the total cell count of CD4^+^CD25^+^ cells in mice did increase^143^. This study may however not be a good comparison to the current *A. phagocytophilum* study, since Fang et al. reasoned that protective immunity against Rickettsia was associated with increased levels of IFN-γ and decreased levels of IL10 in the serum^143^. This notion presented by Fang et al. is in contrast to studies with *A. phagocytophilum* which report a lack of balance between IFN-γ and IL10 as a cause for histopathological lesions in human cases as previously mentioned in the section “Increased upregulation of IFN-γ was prominent between 8 and 14 days after inoculation”.

In the current study, a cytotoxic response by CD8-T-cells is supported by a prominent increase in the essential transcription factor gene *EOMES* for CD8^+^ cells on 8–14 dpi and the upregulation of *CD8a* and *CD8b* on 10–14 dpi (Fig. 7G)^145^. The PBMCs cell count flow cytometry results support the notion of an increased CD8 response during the infection compared with CD4-T-cells, although the total cellular count of CD4^+^, CD8^+^, CD4^+^CD25^+^ and CD8^+^CD25^+^ T-cells showed a wide dispersion and significant alterations were only evident in CD4^+^CD25^+^ T-cells as previously discussed (0–14 dpi)(Fig. 8A–D). However, we observed a significant decrease in the CD4:CD8 ratio in infected sheep at 0–10 dpi (p = 0.0003), 0–14 dpi (p = 0.0223), 3–10 dpi (p = 0.0036), then an increase at 10–14 dpi (p = 0.0429). The skewed ratio between CD4 and CD8- T-cell population imply that CD8 T-cells were more prominent than CD4-cells during the infection (Fig. 8E). Furthermore, ratio shifts in the T-cell populations of sheep infected with *A. phagocytophilum* have been observed previously*,* implying that the shift is a common sequalae in sheep during infection with the bacterium^119, 146^. Still, we would like to emphasize that an increased CD8 T-cell population does not necessarily mean a propagated and functional response against the pathogen. A study by Scorpio et al. showed a reduced production of intracellular IFN-γ in human cytotoxic CD8 T-cells infected with *A. phagocytophilum*^147^.

In the current study, we described immunological pathways and genes that were regulated on crucial time points during infection with *A. phagocytophilum* in sheep. More specific, the study provides a general picture of differentially expressed genes from all cells present in the blood on the time of sampling, which provide a good basis for future studies on transcriptomic data from PBMC or neutrophil granulocytes from infected sheep. However, there are some aspects to consider; First, in our study we analyzed ewes and rams together, since they were in the transition between young and adult based on the hormone levels of testosterone and anti-Müellerian hormone (AMH) shown in the Supplementary (Fig. S4). A previous murine study showed differences in susceptibility against *A. phagocytophilum* with regard to which sex was infected, and it is worth considering if this also occur in sheep^148^. In our study, the AMH-levels in rams were significantly higher than in ewes (p = 0.0079), indicating that the high prepubertal levels of AMH in the rams were declining towards levels observed in adult rams^149^. The levels of AMH observed in the ewes are associated with fertility, indicating that the ewes were becoming sexually mature^150^. The rams showed significantly higher testosterone levels (p = 0.0238) than the ewes (Fig. S4A), although two rams were close to observed levels of the ewes (0.28 and 0.40 ng/ml). Due to the low levels of testosterone in two rams as well as elevated levels of AMH for all the rams, we chose to analyze rams and ewes together in one group in order to highlight the most prominent and important regulation of immunological genes and pathways. Second, the study design is limited to interaction between the host and the bacterium; thus it does not provide information on how the tick may contribute to immune stimulation and immune modulation in host. As previous studies have shown^151–153^, the tick is important in modulating and interacting with the host immune system during transmission with vector borne agents. In future studies it will be important to assess the dynamic interaction between the three agents, the tick, *A. phagocytophilum* and the host in order to achieve a viable vaccine. Moreover, comparing the gene expression data, obtained in this study, with in vitro data in previous studies, may not provide the same picture as both the inoculum and the host animal provide more complexity to the host–pathogen immunological interaction. Future studies should investigate regulation of co-stimulation genes of T and B-cells in secondary lymphoid organs during the infection with *A. phagocytophilum*, in order to develop new strategies for vaccine development against the bacterium.

## Conclusion

To conclude, our results suggest that sheep infected with *A. phagocytophilum* mount a strong innate inflammatory response, followed by a more restrained and suboptimal adaptive immune response. The reasons for the suboptimal adaptive immune response must be investigated and future prospects should focus on understanding the dynamics and mechanisms responsible for the downregulation of co-stimulatory molecules as well as the low activation and proliferation of CD4-T-cells in the host. These cells are instrumental for the development of a robust adaptive immune response and must function accordingly if a vaccine should provide protection. Without such knowledge, it is challenging to ascertain if failure to protect against *A. phagocytophilum* is due to a redundant vaccine antigen or due to a reduced CD4-T-cell response.

### Supplementary Information

Supplementary Information.

## Data Availability

The data supporting the findings of this paper are available from the corresponding author upon reasonable request.
