# Supplementary material for: Temporal patterns of gene expression in response to inoculation with a virulent Anaplasma phagocytophilum strain in sheep
Source: Sci Rep. 2023 Nov 21;13:20399. doi: 10.1038/s41598-023-47801-6 (PMC10663591; doi:10.1038/s41598-023-47801-6)
Supplement: Supplementary file 1 — Supplementary Information. [file 41598_2023_47801_MOESM1_ESM.docx]

**Supplementary.**

**Table s1. Primer and PCR-settings for amplicon.**

Amplicon 64 bp, probe 5῾-FAM-TGC CTG AAC AAG TTA TG-BHQ1-3῾

Ref. ^1^

1 Henningsson, A. J. *et al.* Detection of Anaplasma phagocytophilum in Ixodes ricinus ticks from Norway using a realtime PCR assay targeting the Anaplasma citrate synthase gene gltA. *BMC Microbiol* **15**, 153, doi:10.1186/s12866-015-0486-5 (2015).

| **Target** | **Forward primer** | **Reverse primer** | **Size (bp)** | **PCR-setting** |
| --- | --- | --- | --- | --- |
| *gltA* | 5’TTT TGG GCG CTG AAT ACG AT’3 | 5’TCT CGA GGG AAT GAT CTA ATA ACG 7’3 | 64 bp | Reference Henningson et al. (2015) |

**Table s2. Overview of mAbs used for flowcytometric phenotyping of PBMCs**

Antibodies with superscript ^1^ are primary antibodies, and antibodies with superscript ^2^ are secondary. Producers are listed in regard to superscript; ^1^. Monoclonal antibody center, Washington State University, USA. ^2^ Southern Biotech, AL, USA.

| Antibodies | Clone | Isotype | Final concentration (µg/mL) |
| --- | --- | --- | --- |
| CD4^1^ | GC1A | IgG2a | 5 |
| CD8^1^ | CACT80c | IgG1 | 5 |
| CD25^1^ | LCTB2A | IgG3 | 5 |
| Allophycocyanin (APC) conjugated goat anti-mouse IgG1^2^ |  |  | 2.5 |
| Fluorescein isothiocyanate (FITC-) conjugated goat anti-mouse IgG2a^2^ |  |  | 10 |
| Phycoerythrin (PE)-conjugated goat anti-mouse IgG3^2^ |  |  | 2.5 |

**Fig. s1.** **Clinical, hematological and bacterial load data from control sheep.** A. The median of temperature observations (closed pentagon) (measured against left Y-axis). Open pentagons represent bacterial load on day 0, 6 and 10 (measured against right Y-axis). B. The median of lymphocyte measurements (five-pointed star). The median of neutrophil granulocyte observations (star). Coloured areas represent IQR for the different parameters. Black stippled line in Fig. A describes upper limit for normal rectal temperature in sheep. Green stippled line and purple stippled in Fig. B describe the limit of neutropenia (0.7x10^9^ cells/L) and lymphocytopenia (2.0x10^9^ cells/L) in sheep.


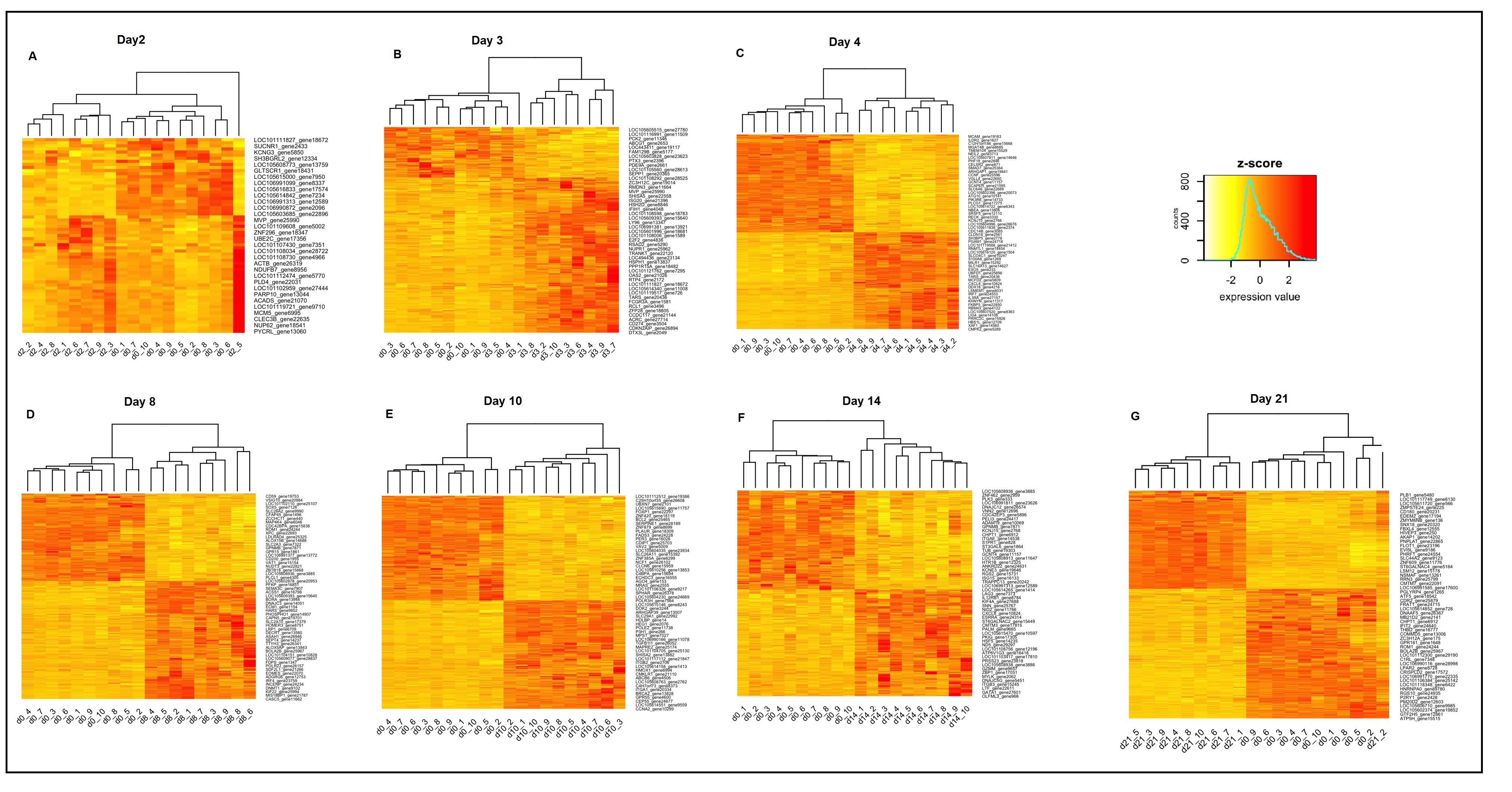


**Fig. s2. Heat-map of DEGS for the different sheep on all days.** A. Day 2. B. Day 3. C. Day 4. D. Day 8. E. Day 10. F. Day 14. G. Day 21


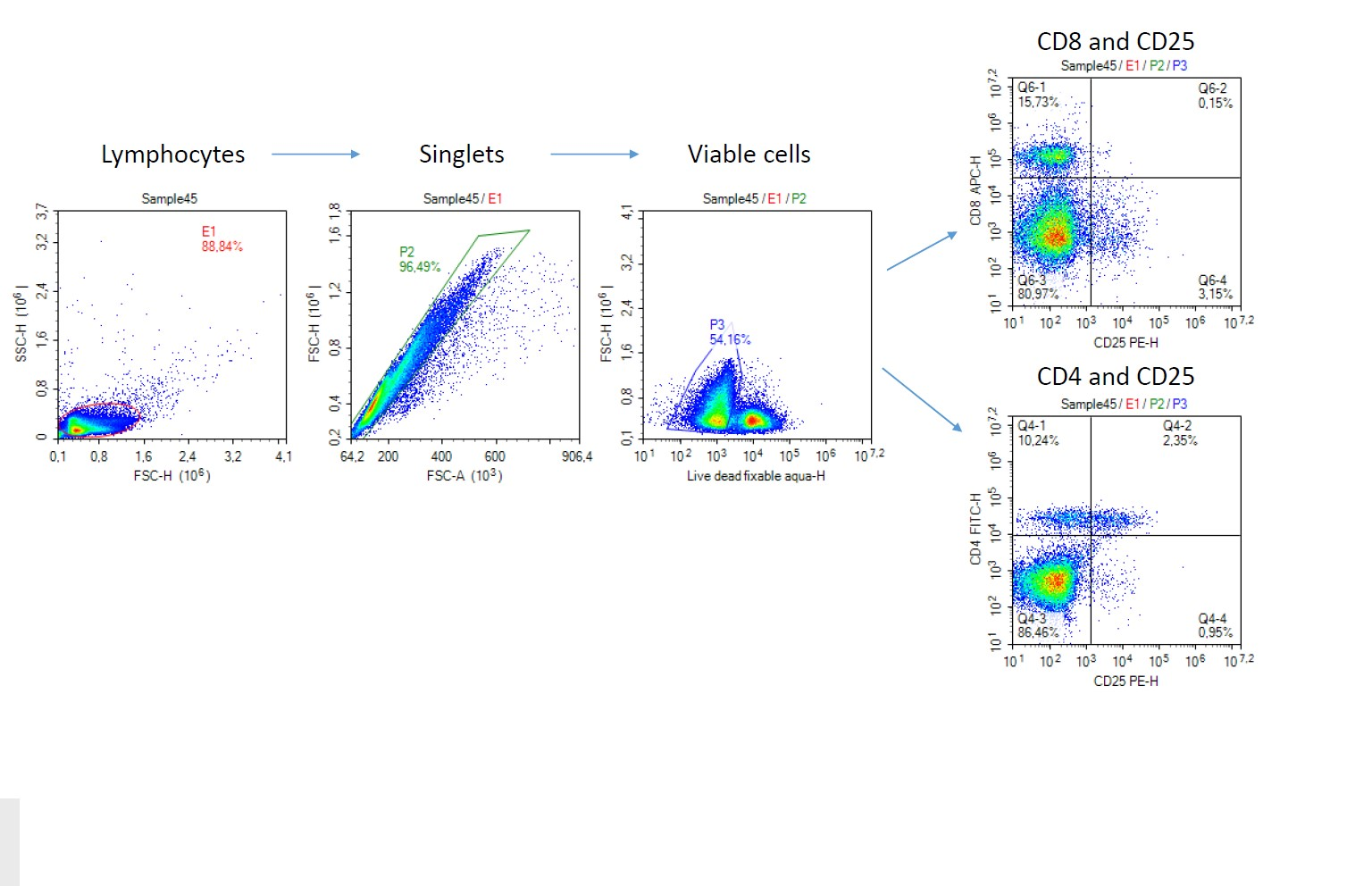


**Fig. s3. Gating strategy for PBMC.**

**Fig. s4. Testosterone and AMH-level in sheep serum.** Serum levels of testosterone (Fig. s4A., circles) and AMH (Fig. s4B., squares) in rams and ewes on 4 dpi. are presented in a box-plot. Individual observations are represented by either circles (testosterone) or squares (AMH), range is determined from min-max. observed values. Non-parametric test were made with Mann Whitney test in GraphPad Prism 10.02 (232). * p=0.0238, ** p=0.0079.
